# Expansion of HIV Laboratory Diagnostic Services in Chennai, India 2001–2006: Is the Growth Commensurate with the Need?

**DOI:** 10.1371/journal.pone.0003471

**Published:** 2008-10-21

**Authors:** A. K. Srikrishnan, Kartik K. Venkatesh, Suniti Solomon, Easter Thamburaj, S. Anand, K. G. Kosalaraman, Pachamuthu Balakrishnan, Kenneth H. Mayer

**Affiliations:** 1 YRG CARE, VHS, Chennai, Tamil Nadu, India; 2 Miriam Hospital, Division of Infectious Diseases, Brown University Medical School, Providence, Rhode Island, United States of America; National AIDS Research Institute, India

## Abstract

**Objective:**

To describe the changes in HIV services provided and the patient population utilizing voluntary counseling and testing (VCT) services at private testing laboratories in Chennai, India in 2001 and 2006.

**Methods:**

In 2001, a cross-sectional descriptive survey was conducted to assess the services provided and client population of 1,031 private laboratories. A subset of labs (9%) that had been surveyed in 2001 were also studied in 2006.

**Results:**

In 2001, significantly more high volume labs (>10 HIV tests per month) offered HIV diagnostic tests than low volume labs (<10 HIV test per month) (p<0.001). More high volume labs (20.0%) provided pre-test counseling as part of HIV testing than low volume labs (11.1%) (p = 0.003). Between 2001 and 2006, the number of labs that provided HIV diagnostic tests significantly increased, including ELISA (87.8% vs. 40.0%), Western Blot (84.4% vs. 13.3%), and Tridot (98.9% vs. 72.2%) (p<0.001). Also the number of labs that reported greater than 10 women seeking HIV testing per month significantly increased from 14.5% to 79.0% (p = 0.006). More labs provided pre-test counseling in 2006 (34.4%) than in 2001 (21.1%) (p = 0.046).

**Conclusions:**

Though HIV diagnostic testing services have increasingly become available, counseling services have not expanded commensurately. Further outreach and education is necessary to expand comprehensive HIV VCT services in both urban and rural India.

## Introduction

It is estimated that 2–3 million Indians are currently living with HIV and the overall HIV prevalence continues to rise [1]. Tamil Nadu in South India is one of the six Indian states which has been classified as high prevalence, defined as having rates in excess of 5% among high risk groups and in excess of 1% of antenatal women [1], [2]. Although the epidemic in India began in high-risk populations, such as female sex workers, truck drivers, and intravenous drug users, infection has now spread into the general population, both urban and rural [3], [4]. Increasingly married monogamous women have presented with a high prevalence of HIV in India, suggesting a strong risk from their spouses [5].

Voluntary Counseling and Testing (VCT) is recognized as an integral element of any effective HIV public health primary prevention and care program [6]. The process of finding out one's HIV status, regardless of the test result, offers a critical opportunity for education and prevention aimed at reducing the risk of HIV transmission. For individuals found to be infected with HIV, VCT can provide a means to accessing further services, including counseling support, antiretroviral therapy (ART), and medical care. Studies in the developed and developing world have demonstrated that VCT can reduce high risk sexual practices [7], [8], [9] and can be a cost-effective intervention [10]. HIV voluntary counseling and testing (VCT) has increasingly become a central prevention strategy in the national AIDS control policies of most developing countries as it serves as an important entry point into care [9].

In India, VCT has been available in certain locations since 1986 [11], and has been widely implemented as part of the HIV prevention program of the National AIDS Control Strategy since 1999 [12]. As low-cost antiretroviral therapy has increasingly become available in India [13], VCT has become an important link connecting individuals to treatment and care. However, though VCT services have become available in India [14], accessing these services has been hampered by a lack of resources to fully implement VCT programs and societal stigma associated with HIV. Multiple studies in other settings have documented the barriers that hinder individuals from accessing VCT services, including denial of HIV risk, fear of stigma, discrimination, disease, and death [7], [15], [16].

Recent studies in India have demonstrated that ongoing counseling and testing can lead to reduced risk taking behaviors and increased condom use[17]. A study at our center examined the acceptability of prenatal HIV testing among pregnant women in which 86% of women were willing to accept VCT [18]. Another recent study at our center examined the different reasons why men and women attended VCT services [19]. In this study, we describe the changes in services provided and the patient population utilizing VCT services at private testing laboratories in Chennai, India in 2001 and 2006. Understanding the availability of current VCT services offered by private laboratories has implications for the further development of education, outreach, and other HIV prevention services.

## Methods

### Setting

The study was conducted by YRG Centre for AIDS Research and Education, a large tertiary HIV care facility in South India [13], [20]. YRG CARE provides comprehensive care and support services to over 10,000 HIV-infected persons, including anonymous voluntary counseling and testing (VCT), integrated medical services for the treatment of HIV and related illnesses, prevention programs, and nutrition education. There is no formal advertising about YRG CARE or VCT services; however, the organization's activities are often featured in the local and regional news and VCT services are discussed as part of YRG CARE's educational and outreach services.

### Survey

In 2001, a cross-sectional descriptive survey was conducted to assess the services provided and client population of 1,031 private laboratories in Chennai, the capital of Tamil Nadu state in southeastern India with a population of over 7 million. The Chennai metropolitan area was divided into eight zones in which field workers identified private laboratories street by street. Hospitals, nursing homes, and other clinical facilities that may also provide laboratory testing services were also identified within each zone. All consenting laboratory testing facilities were then administered the survey. Labs that performed greater than 10 HIV tests per month in 2001 were defined as high-volume facilities (N = 140), and those with less than 10 HIV tests per month in 2001 were defined as low-volume facilities (N = 891). A subset of labs (9%) that had been surveyed in 2001 were also studied in 2006. This study was conducted among privately operated labs because the primary source of healthcare for Indians has been private facilities.

The survey assessed the current availability of basic clinical testing services, STD testing, HIV testing, counseling services, and willingness to build skills in offering VCT services. The survey was administered by outreach staff to consenting lab personnel who provided written consent to take part in the survey. This study received institutional review board (IRB) approval at both the free-standing YRGCARE IRB and the Brown University-affiliated Miriam Hospital IRB.

### Statistical Analysis

Descriptive statistics were used to calculate the frequency, mean, median, and standard deviation (SD). Diagnostic tests were performed, and the mean+/−SD was used for variables that were normally distributed; and the median and interquartile range (IQR) were calculated for variables that were influenced by extreme variables. To compare proportions, chi-square (χ^2^) statistics and independent t-tests were used. A p value of less than 0.05 was considered statistically significant. Data entry, database management, and statistical analyses were performed with SPSS software (version 13.0; SPSS, Chicago, IL).

## Results

In 2001 (N = 1031), 64.7% of survey respondents were lab technicians, 20.4% were owners/directors, and 9.2% were physicians; the breakdown of respondents was similar in 2006. The median number of years of operation was 6 years (IQR: 3–12) for low volume labs and 10 years (IQR: 5–17) for high volume labs. The median number of employees was 2 personnel (IQR: 1–3) for low volume labs and 5 personnel (IQR: 2–10) for high volume labs. The mean length of time counselors had been with the lab was 0 years for low volume labs and 1.4 years for high volume labs. In 2001, labs offered the following basic testing services: 68.6% liver function testing, 95.5% hemoglobin, 81.1% complete heamogram, 93.8% renal function testing, 92.1% cholesterol, and 81.0% triglycerides; there were modest increases in these basic testing services in 2006 (N = 90).

In 2001, significantly more high volume labs offered HIV diagnostic tests than low volume labs, including ELISA (59.3% vs. 20.0%), Tridot (79.3% vs. 49.6%), and Western Blot (15.0% vs. 2.5%) (p<0.001) (See Table 1). Significantly more high volume labs reported more than ten men (41.4%) and ten women (39.9%) accessing their facilities for HIV testing in the last month than low volume labs (0.7% men; 0.4% women) (p<0.001). More high volume labs (20.0%) provided pre-test counseling as part of HIV testing than low volume labs (11.1%) (p = 0.003).

**Table 1:** HIV testing and counseling services in 2001 among low volume (<10 HIV tests per month) and high volume laboratories (>10 HIV tests per month) (N = 1031).

	2001 Labs <10 HIV tests per month (Mean, %)	2001 labs >10 HIV tests per month (Mean, %)	p-value
	N = 891	N = 140	
**HIV Testing services**			
**HIV diagnostic tests offered by labs**			
ELISA	20.0	59.3	<0.001
Western blot	2.5	15.0	<0.001
Tridot	49.6	79.3	<0.001
**Lab conducted HIV testing in last month**	59.0	100.0	<0.001
**Males who came for HIV testing in the last month**			<0.001
>5	42.8	25.0	
6–10	4.5	25.7	
>10	0.7	39.9	
**Females who came for HIV testing in the last month**			<0.001
>5	39.5	14.3	
6–10	4.5	34.3	
>10	0.4	41.4	
**HIV Counseling Services**			
**Lab provided pre-test counseling**	11.1	20.0	0.003

Between 2001 and 2006, the number of labs that provided HIV diagnostic testing significantly increased, including ELISA (87.8% vs. 40.0%), Western Blot (84.4% vs. 13.3%), and Tridot (98.9% vs. 72.2%) (p<0.001) (See Table 2). In 2006, all of the surveyed labs performed greater than 10 HIV tests per month, while in 2001, 42.2% of these labs provided greater than 10 HIV tests per month (p<0.001). Between 2001 and 2006, the number of labs that reported greater than 10 women seeking HIV testing per month significantly increased from 14.5% to 79.0% (p = 0.006).

**Table 2:** Changing patterns in HIV testing, counseling, and clinical services from 2001 to 2006 (N = 90).

	Labs in 2001 (Mean, %)	Labs in 2006 (Mean, %)	p-value
	(N = 90)	(N = 90)	
**HIV Testing Services**			
**HIV diagnostic tests offered by labs**			
ELISA	40.0	87.8	<0.001
Western blot	13.3	84.4	<0.001
Tridot	72.2	98.9	<0.001
**Reasons clients sought STD/HIV testing**			<0.001
Job related	91.1	70.0	
Migration	8.9	27.8	
**Number of HIV tests performed in a month**			<0.001
<5	20.0		
6–10	23.3		
>10	42.2	100.0	
**Males who came for HIV testing in the last month**			NS
<5	36.7	16.7	
6–10	16.7	20.0	
>10	22.3	31.1	
**Females who came for HIV testing in the last month**			0.006
<5	34.4	5.6	
6–10	15.6	10.0	
>10	14.5	79.0	
**Number of HIV tests that were physician referred**			<0.001
<5	31.1	4.4	
6–10	21.1	10.0	
>10	30.0	78.9	
**Number of tests that were walk-ins**			0.034
<5	32.2	20.0	
6–10	6.7	8.9	
>10	5.5	14.4	
**HIV Referral Services**			
**Source of physician referrals**			<0.001
Physicians in the same area	75.6	52.2	
Physicians from elsewhere	5.6	37.8	
**Client referred to physician or institution when testing positive for HIV**	56.7	40.0	0.025
**HIV Counseling Services**			
**Lab provided pre-test counseling**	21.1	34.4	0.046

The number of labs receiving greater than 10 physician referrals for HIV tests significantly increased between 2001 (30.0%) and 2006 (78.9%) (p<0.001); and the number of labs receiving greater than 10 walk-ins for HIV testing also significantly increased between 2001 (5.5%) and 2006 (14.4%) (p = 0.034). From 2001 to 2006, labs increasingly received referrals from physicians not in the same area as the lab facility (5.6% to 37.8%) (p<0.001). In 2006, significantly fewer labs (40.0%) referred clients testing positive for HIV to a physician or another institution than in 2001 (57.7%) (p = 0.025). A small minority of labs (<5%) referred clients testing positive for HIV to alternative medical providers both in 2001 and in 2006. More labs provided pre-test counseling in 2006 (34.4%) than in 2001 (21.1%) (p = 0.046).

Between 2001 and 2006, more labs reported that the reason clients sought HIV testing was due to migration (8.9% vs. 27.8%), and fewer labs reported job related reasons (91.1% vs. 70.0%) (p = 0.001). In 2001, more high volume labs (60.7%) expressed interest in having a staff member trained in counseling than low volume labs (38.3); however among the 90 labs assessed in both 2001 and 2006, fewer labs (6.7%) in 2006 were interested in having a staff member trained in counseling than in 2001 (58.9%).

In addition to an increase in performing HIV testing, assays for other common pathogens significantly increased from 2001 to 2006: 87.8% to 100% offered screening tests for syphilis (RPR/VDRL); 32.2% to 73.3% syphilis confirmatory testing, 31.1% to 81.1% Trichomonas wet mount, 74.4% to 97.8% Hepatitis B Surface Antigen (HBsAg), and 73.3% to 86.7% acid fast bacilli (AFB) smear (p<0.005).

## Discussion

This study demonstrates that a considerable number of private laboratories in Chennai currently have the infrastructure to provide comprehensive STD and HIV testing services with a high volume of clients. Though in India public awareness and knowledge of HIV and AIDS remains relatively low [17], the labs in 2006 appear to have expanded their testing services since 2001, which may be reflected in increasing numbers of women seeking HIV testing, possibly as a result of recent regional efforts to increase antenatal testing. Labs performed a range of HIV testing services, with almost all labs providing ELISA, Western Blot, and Tridot, for their clients in 2006. Most labs followed Indian government National AIDS Control Organization (NACO) guidelines[21], which involved using a test with very high sensitivity followed by a second test with very high specificity. These tests generally include a rapid test, which can involve ELISA or Tridot-based technologies. In cases of indeterminate results, a Western Blot is used as a confirmatory test. However, most of these labs lack policies and procedures for quality assurance and quality control (QA/QC). The number of labs performing ELISA more than doubled and Western Blot increased by over six fold between 2001 and 2006, suggesting an increasing level of sophistication in HIV testing services.

Increasing efforts in India have been underway to encourage general physicians to order HIV testing along with other routine lab tests [22]. The majority of referrals to the surveyed labs came from physicians, and most of the physicians who made these referrals were located within the vicinity of the lab facility. It has been suggested that a major barrier to seeking VCT services in other resource-poor settings is logistical, including location of the testing center [16]. In light of the increasing availability of HIV testing services throughout Chennai in this study, it is probable that clients can conveniently access labs within their localities. However, it is estimated that 90% of HIV-infected Indians are unaware of their status [23], raising concerns that stigma and ignorance may be impeding wider access to testing.

Over three fourths of labs reported over ten women seeking HIV testing in a month in 2006 compared to 2001 when less than a fourth of these same labs reported over ten women seeking HIV testing in a month. In contrast, only close to a third of labs in 2006 reported more than ten men seeking HIV testing in a month. It is possible that increasingly Indian women are seeking HIV testing services at private laboratories as part of antenatal screening. Also, most Indian men may be diagnosed with HIV after presenting to clinical care with an opportunistic infection, and their asymptomatic wives may then be consequently tested for HIV[4], [5], [19], [24]. Indian women have reported seeking VCT services as part of prenatal testing to protect their baby's welfare [18]. Though some Indian women may be willing to be tested, others may be concerned about confidentiality and disclosing HIV serostatus out of fear of negative reactions from their husbands, parents, and community [25]. As more women seek VCT services, counseling approaches will need to be developed that ensure confidential couple/family based counseling.

The labs surveyed in 2001 and 2006 reported that the reasons clients sought HIV testing was due to migratory and job-related reasons. Though the number of labs reporting migration significantly increased from 2001 to 2006 relative to job-related reasons, it is very likely that job-related reasons are the same as migratory reasons, as both reasons provide documentation necessary for Indians to seek work overseas. Currently, there is no mandatory HIV testing for domestic employment neither in the Indian public nor private sectors. However, the Gulf States, which have a large number of South Asian nationals working as guest workers, have required mandatory HIV testing of all foreign nationals since the 1990s [26], [27]. Before being granted a resident permit, foreign nationals entering the Gulf States are tested in their country of origin. The reasons individuals sought VCT services at the private labs in this study differed from prior self-report studies at YRG CARE, which found that the major reason men sought testing was due to risk behavior and HIV-related symptoms and the major reason for women was a HIV-seropositive partner [19], [28]. Based on the current study, individuals utilizing HIV testing at these labs are not seeking these services due to their own risk profile or symptoms, but due to immigration requirements. New approaches to testing and accessing at risk individuals must be developed before HIV infected individuals report with opportunistic infections for clinical care.

Fewer labs in 2006 referred their clients testing positive for HIV to external physicians or institutions than in 2001. It is likely that more private labs provide in-house HIV care and treatment at affiliated institutions, however studies have shown that private Indian institutions may lack adequate training in providing HIV services [29], [30]. Additionally, less than a tenth of labs were interested in training lab personnel in counseling, while in 2001, over half the labs were interested in training lab personnel. By 2006, an increasing proportion of labs may have developed their own systems of counseling as part of offering more comprehensive testing services, and thus no longer required training for their staff. Though more labs offered counseling services to clients seeking HIV testing in 2006 than in 2001, only a third of labs had a system of counseling in place, either involving a permanent in-house counselor or a counselor that could be contacted on as-needed basis. HIV testing without appropriate follow-up counseling can pose risks to the physical, emotional, and psychological functioning of the client, especially within an Indian setting where AIDS-associated stigma is great. South Indian women have reported that a positive HIV test result included thinking you would die soon, suffering from psychological stress, and wanting to kill/hurt themselves [25]. With the high number of labs providing HIV rapid testing in this study, same day pretest and posttest counseling could be implemented.

Though HIV testing services at the most experienced private laboratories have considerably expanded from 2001 to 2006, there is still a substantial underutilization of counseling services as part of HIV testing. As most of the assessed labs are structured primarily as testing centers that receive clients via physician referral, these facilities may not have experience in providing client-centered counseling. Health counseling itself is a relatively new concept in India where openly discussing sexual lifestyles is often taboo and patients may not be proactive in seeking health care services [14]. The disparity between centers providing HIV testing without commensurate voluntary pre- and post-testing counseling (VCT) suggests the increasing need for massive professional educational efforts of healthcare providers and lab personnel.

In the context of a lack of adequate universal HIV/AIDS treatment, there are potentially many perceived deterrents to being tested for HIV which can outweigh the benefits of knowing one's status. However it is possible with the increasing availability of ART, more individuals may seek VCT services [19]. Not seeking VCT services may also reflect social barriers to getting tested, especially AIDS-related stigma, rather than a lack of a perceived value of getting tested [7]. Within the Indian medical establishment, individuals may be routinely tested for HIV without consent before undergoing elective surgery or delivery, and if found to be HIV infected may be refused care. Innovative models have been proposed for increasing uptake of VCT services in other settings, such as same-day rapid testing via a mobile VCT approach[16] and same-day results using rapid test kits, anonymized recordkeeping, lay counselors, and peer educators [15].

As the current study documents, VCT services have now become available in urban settings, but increasingly as HIV spreads to rural India, VCT services must also geographically expand. As developing countries, including India, are increasingly able to provide antiretroviral treatment, VCT will become an important tool for linking people to care. The current study showed an increased use of HIV rapid testing with same-day results, which may allow more individuals to receive their test results. It has been suggested that the cost-effectiveness of VCT services can be achieved through supporting VCT services in high-prevalence areas, by linking VCT services to reach high-prevalence populations, and by targeting individuals who are or were sexual contacts of known HIV-infected individuals [10]. In India, public health efforts to create more comprehensive VCT programs can be more effective by acknowledging and involving private clinical laboratories. Future national expansions of offering increased VCT services must develop methods of involving the Indian private sector, which is likely to remain the largest provider of HIV testing for the foreseeable future.
